# Prescriptions and Dispensing

**Published:** 1908-04-11

**Authors:** 


### Therapeutics.

### PRESCRIPTIONS AND DISPENSING.

### PILLS.—I.

Notwithstanding the many forms of medicine of comparatively recent introduction, such as cachets, capsules, tablets, tabloids, bipalatinoids, and so forth, pills easily take the next place after mixtures in importance. By far the greater part of the pills produced to-day are made in large quantities by machinery, and reach the public through the chemist.

There are still many doctors, however, who make their own pills; and it not infrequently happens that a special pill may be required for some particular purpose—a pill that is not made wholesale. When this is so, the doctor either makes the few pills required himself, or else sends a prescription for them to a chemist who is to make them up. It is only the more or less common pills that can be made wholesale; unusual pills are made for each particular occasion, and it is amongst these that difficulties are liable to arise.

Before discussing the difficulties in detail, and the methods of overcoming them, we may recall the objects which are to be aimed at, and which are the same for pills as for mixtures. These, as we have stated in a former article, are:—

(1) Preparation of the medicine in a form in which it can be readily absorbed or assimilated. (2) Even distribution of the medicaments amongst the doses. (3) The prevention of mutual decomposition or reaction among the different ingredients if they are liable to such change, except in those cases in which the reaction is foreseen and especially desired.

Applying these general principles to the case of pills, we see that (1) requires the pills, and the coating, if there be any, to be easily dissolved or disintegrated in the stomach; (2) requires that the pill-mass shall be homogeneous and divided into pills of equal weight. Besides these therapeutic requirements, we must consider the demands of elegant dispensing, which requires that the pills shall be as agreeable to look at and to swallow as possible; for example, they should not be larger than is absolutely essential, they should be well rounded, and they should retain their shape when kept under fair conditions.

It is sometimes the case that the particular medicaments prescribed in a pill are of such a nature that by merely mixing them and working them well together with the pestle, a mass results which is suitable for rolling and dividing into pills. This, however, is quite an exception; as a rule it is necessary to add some inert ingredient or ingredients in order to make such a mass. This inert addition is known as the excipient, and doctors who write prescriptions to be made up by a chemist often leave to the latter the selection of the excipient and the adjustment of the quantity of it that should be used. Even when the prescription includes an excipient, it is recognised that a chemist is within his rights in altering it if there is any reasonable occasion for doing so. Doctors who make their own pills usually favour one excipient or another, each using his favourite in the case of most of the pills he makes up.

The active ingredients, however, may make it advisable to use one excipient in one case, another in a second. The ingredients may be powders or substances that can be powdered, or even partly powders and partly liquids, such as essential oils, which, when mixed, form a dry or nearly dry material, and in such a case the excipient must be something which will bind the powder into a mass. On the other hand, the ingredients may be wholly or in part extracts, or liquids, so that when mixed a fluid or semi-fluid material results; in this case the kind of excipient and manipulation required will be the reverse of what they were in the former case.

While the number of substances that may occasionally be required as excipients is large, a small number suffices for most ordinary pills. No hard and fast rules can be given as to which should be used, as in a great many cases several are equally good. *For binding dry powders* the principal excipients are:—Glycerin of tragacanth, confection of roses, malt extract, simple syrup, syrup of glucose, mucilage of acacia.

*For dry extracts or other powders of a sticky nature*:—Water, spirit, dilute glycerin.

*For masses that are already too soft*:—Powdered liquorice, marsh mallow, tragacanth gum.

Soft masses may be stiffened by drying, which is often better, as we shall see, than adding powder; on the other hand, liquorice or marsh mallow must sometimes be added, not as drying agents only, but on account of their fibrous nature. Pills to which some such fibrous substance has been added often retain their shape better than they do without it.

The art of making good pills involves a good deal more than merely selecting the right excipient; proper familiarity with the necessary manipulation can only be obtained by experience. Generally speaking, a good deal of "elbow-grease" is necessary. An amount of excipient which, gently stirred into a powder, would barely suffice to make it sticky, may be ample to make it a soft mass if it is well worked in. This is no doubt partly due to the fibres in the powder being squeezed closer together, and a larger quantity of them therefore becoming coated with a given quantity of the sticky material present, but it is also partly the result of the heat generated by the friction, which softens ingredients of an extract of resinous nature, and so helps them to spread further.

The mass should be rolled out and cut into pills as soon as it is made, and the pills at once rounded off. After a few minutes a well-made mass will in most cases become appreciably harder, and it is best for this only to occur after the pills have been rolled.

Manipulation of the mass with the fingers is often so advantageous, or even necessary, that it cannot be objected to. It is hardly needful to add that the hands must be washed absolutely clean before pill-making.

